# Orbital mucinous tumor and systemic multiple mucinous tumors: Radiological analysis, differential diagnosis, and case report

**DOI:** 10.1097/MD.0000000000043702

**Published:** 2025-08-01

**Authors:** Wen Gao, Zhao Li

**Affiliations:** a Ophthalmic Center, 474 Hospital of Xinjiang, Urumqi, Xinjiang Uygur Autonomous Region, China; b Ophthalmology Department, General Hospital of Xinjiang Military Region, Urumqi, Xinjiang Uygur Autonomous Region, China.

**Keywords:** characteristics, pathological, radiological, surgery

## Abstract

**Rationale:**

Orbital mucinous tumors are exceptionally rare, with multifocal systemic involvement posing significant diagnostic and therapeutic challenges. This study aims to characterize the radiological and pathological features of such cases, refine differential diagnosis criteria, and evaluate treatment strategies through a comprehensive analysis of a rare bilateral orbital mucinous tumor with synchronous pulmonary and retroperitoneal lesions. Our findings aim to bridge gaps in understanding this rare disease’s behavior and management.

**Patient concerns:**

We retrospectively analyzed the medical history, examinations, surgeries, and pathological findings of a case involving multiple intraorbital cysts combined with multiple systemic organ cysts.

**Diagnoses:**

We studied the characteristics, progression, and variations of such rare diseases to better understand their diagnostic and therapeutic strategies.

**Interventions:**

The patient underwent general anesthesia for bilateral orbital and eyelid incisions with conjunctival access. Multiple cystic masses of varying sizes were removed, with the masses adherent to the superior orbital nerve.

**Outcomes:**

Upon incision, yellowish fluid was drained, and pathological examination confirmed the masses as mucinous tumors.

**Lessons:**

Orbital mucinous tumors are extremely rare, and cases combined with multiple systemic involvement are even rarer. Radiological examinations provide clarity on cyst density, while pathological examination further elucidates the nature of the masses. Surgery remains the primary treatment modality, with postoperative follow-up being crucial.

## 1. Introduction

Mucinous tumors are rare benign lesions characterized by mucin-rich stroma containing stellate spindle cells.^[1]^ While these tumors can occur in various anatomical sites, orbital involvement remains exceptionally uncommon. We present a rare case of bilateral orbital mucinous tumors with synchronous pulmonary and retroperitoneal involvement, with 3 key objectives: to characterize its imaging and pathological features, to analyze differential diagnoses using current criteria, and to investigate optimal treatment strategies through comprehensive case analysis.

## 2. Background

First described by Virchow in 1871, mucinous tumors were later defined by Stout in 1948 as stromal tumors featuring undifferentiated stellate/spindle cells in a mucoid matrix.^[2]^ Though most commonly found in skin and subcutaneous tissue, they may rarely occur in the orbit, possibly originating from embryonic mucoid tissue remnants. Orbital cases, first reported by Krueger in 1967, typically show benign histology with slow growth, though local infiltration and rare malignant transformation have been documented.^[3]^ Their characteristic histology reveals a mucoid matrix with delicate collagen fibers and stellate cells resembling mesenchymal precursors.

This case highlights both the diagnostic challenges of multifocal mucinous tumors and the importance of multidisciplinary collaboration in orbital pathology.

## 3. Case report

**Figure 1.**
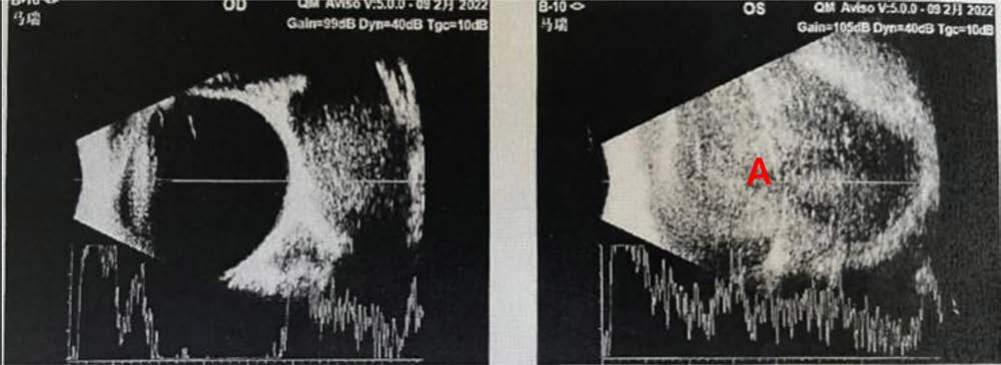
In the upper left orbit, a roughly 25.77 mm × 34.02 mm hypoechoic area is observed with relatively clear borders and uneven internal echoes (A).

**Figure 2.**
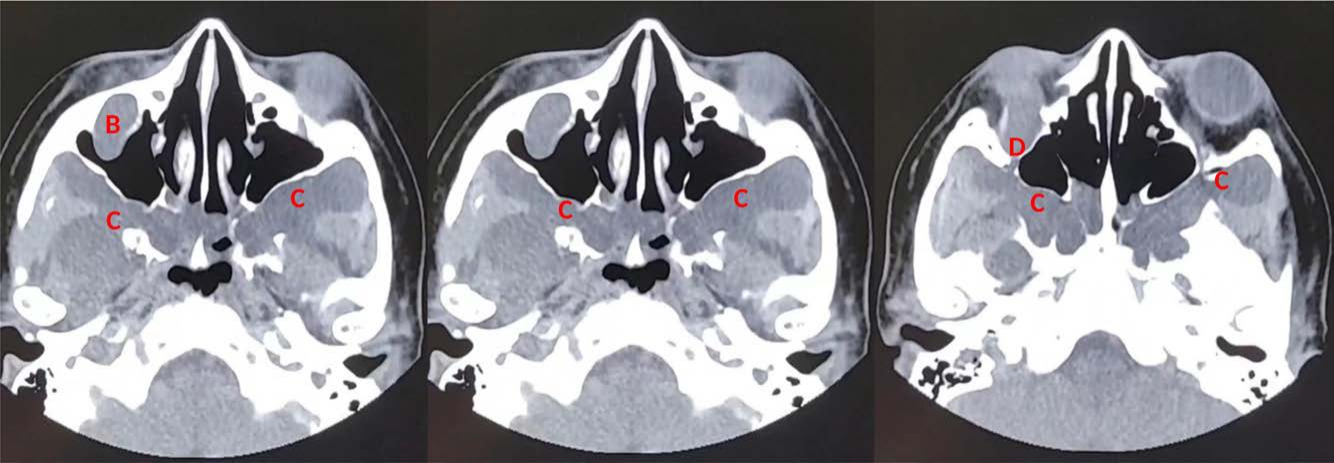
The axial images of the orbital CT reveal lesions characterized by low-density shadows (B), extensively involving the right infraorbital canal, bilateral nasopharynx, infratemporal fossa, pterygopalatine fossa (C), and the muscle spaces in the middle cranial fossa. Additionally, there is enlargement of the right infraorbital canal (D). CT = computed tomography.

**Figure 3.**
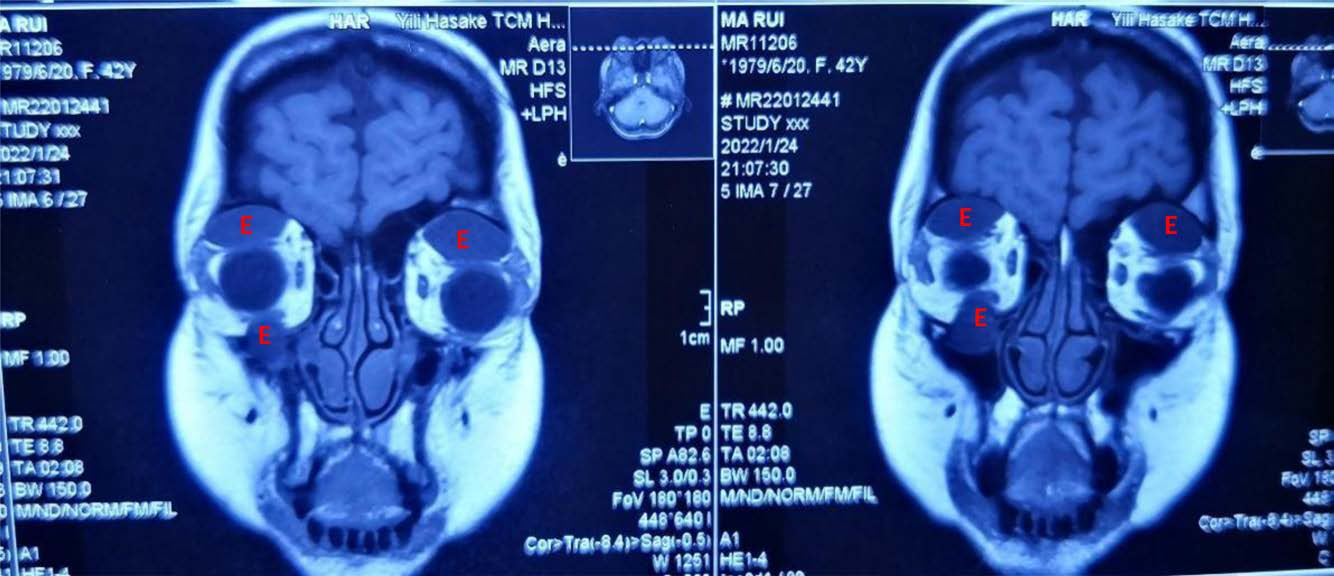
The MRI of the orbits reveals on the TI-weighted sequence, bilateral upper orbital regions and the right infraorbital nerve canal area show cluster-like slightly low signals (with slightly prolonged TI) (E). MRI = magnetic resonance imaging.

Patient is a 42-year-old female of the Hui ethnic group presenting with the chief complaint of “left eye swelling for more than 3 months.” Since early December 2021, she noticed an elliptical peanut-sized swelling above the left eyeball without apparent cause or provocation. It is non-tender. She visited our hospital outpatient department where orbital computed tomography (CT) scan revealed multiple intraorbital lesions involving the maxillary and infratemporal fossa. The patient was admitted for further evaluation of the orbital mass lesions. In the past, she underwent excision of a left parotid cyst in 2008 at a local hospital, and the postoperative pathology report indicated a benign lesion (specific nature unspecified). On examination: visual acuity: right eye 1.0, left eye 0.5. Proptosis: 22-101-23. Eye movements: Full in all directions in the right eye, partially limited upward gaze with diplopia in the left eye. Right eye examination unremarkable; Left eye shows inferior and lateral displacement, incomplete eyelid closure with partial corneal exposure. Mild conjunctival congestion, whitish band-like opacity beneath the cornea, otherwise no abnormalities noted. Intraocular pressure: 20 mm Hg in the right eye, 18 mm Hg in the left eye. Ocular B-ultrasound (Fig. 1) revealed a roughly 21.27 mm × 11.76 mm hypoechoic area in the lower right orbit with relatively clear borders and uneven internal echoes; in the upper left orbit, a roughly 25.77 mm × 34.02 mm hypoechoic area with relatively clear borders and uneven internal echoes. Optic disc optical coherence tomography showed no obvious abnormalities in optic nerve thickness bilaterally. Orbital CT (Fig. 2) scan showed bilateral proptosis, multiple irregular slightly low-density lesions within the orbits, with CT values of 14 HU and clear margins. Compression of the superior rectus muscle and right inferior rectus muscle, right infraorbital fissure enlargement, bilateral orbital apex enlargement, extension of lesions posteriorly through the inferior orbital fissure into the infratemporal and maxillary fossae, and local bone compression and absorption changes were noted. Orbital MRI (Fig. 3) revealed bilateral proptosis, forward protrusion of both lacrimal glands, oval-shaped abnormal signals of various sizes in the bilateral orbital apex, intraorbital regions, bilateral temporal fossae, pterygopalatine fossae, with low signal on TIWI and high signal on T2WI. Right inferior orbital fissure and bilateral pterygomaxillary fissure widening, abnormal signals of various sizes extending from the temporal fossae through the pterygopalatine fossae to the nasal cavity, adjacent bone compression.

**Figure 4.**
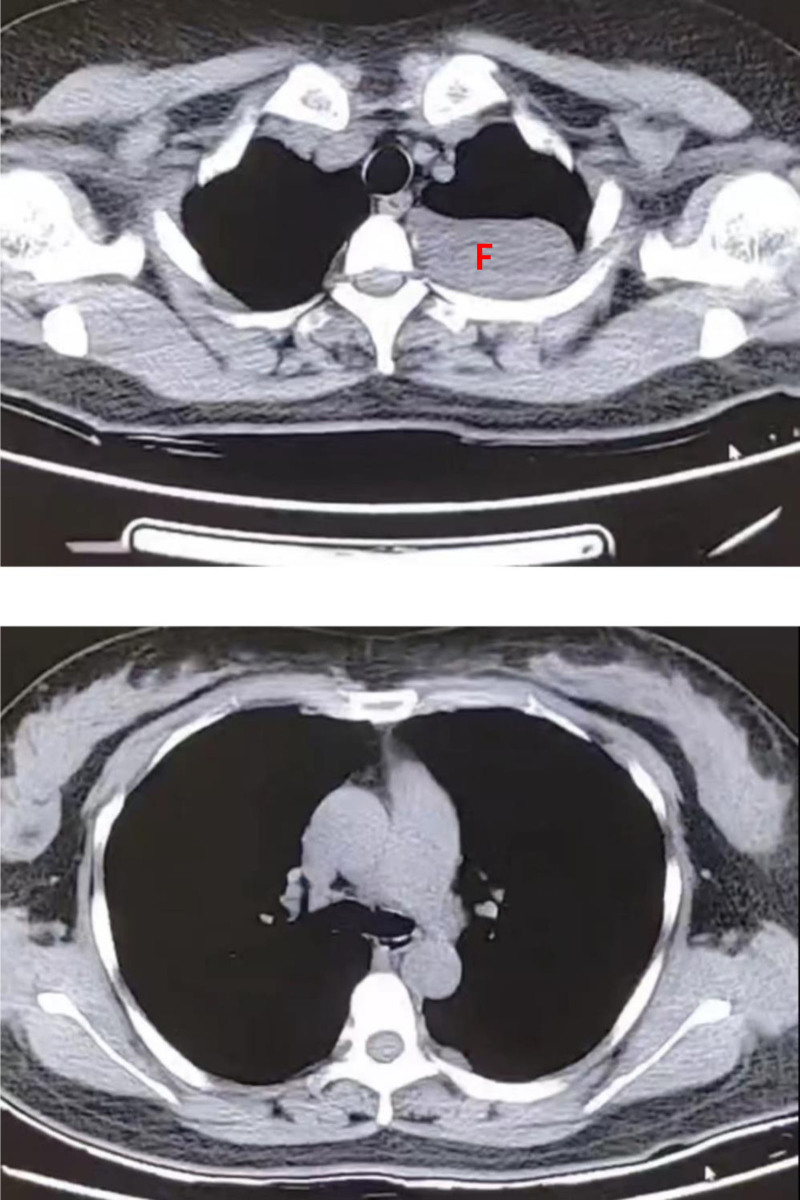
The mediastinal window confirms that the lesion is located beneath the pleura (F), and its sharp angle with the mediastinum indicates that it does not originate from the mediastinum. There are no enlarged lymph nodes in the mediastinum or axilla, and no abnormal changes are observed in the bones.

**Figure 5.**
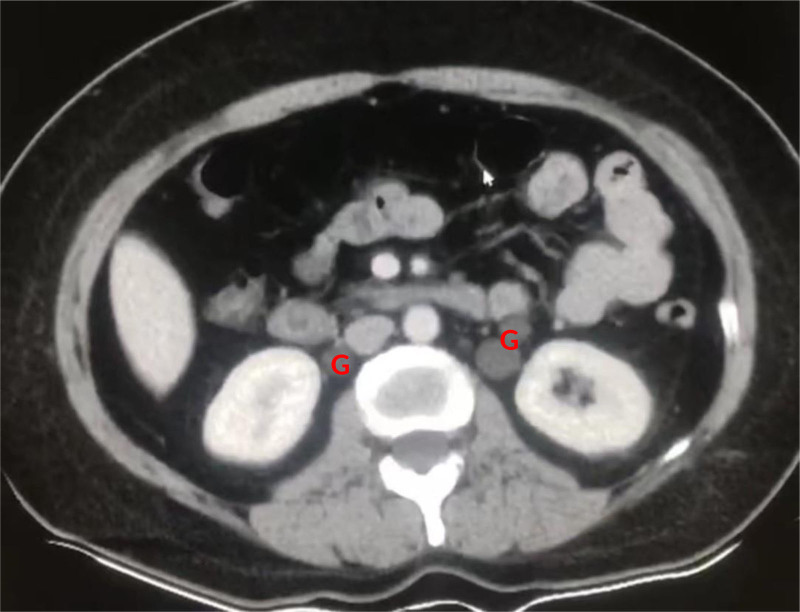
Multiple low-density masses are visible anterior to both kidneys at the level of the renal plane in the retroperitoneum (G). These masses exhibit similar CT density values to those observed in the orbits and chest lesions, and there are no enhancement changes noted after contrast administration. CT = computed tomography.

Based on the current examinations, it is confirmed that the patient has multiple cystic occupying lesions within both eye sockets invading the periorbital tissues. These masses compress the surrounding bones but do not exhibit malignant invasive changes such as erosion, ossification, or lytic lesions. The plan is to perform under general anesthesia the removal of the cysts within both eye sockets, thus further preoperative investigations are being conducted. Preoperative tests, electrocardiogram, and echocardiography show no significant abnormalities. However, a chest radiograph in the lateral view suggests a slightly elevated density mass in the left lung apex, with uniform density and clear margins observed on the lateral film. To mitigate the risk of potential systemic diseases and surgery complications, further refinement of chest CT and contrast-enhanced examinations is underway. The chest CT in lung window reveals elevated density masses beneath the pleura of the left lung apex and left lower lung dorsal region, with clear boundaries, smooth edges, and uniform density, and adjacent structures show no apparent abnormalities. In the mediastinal window (Fig. 4), the lesion is confirmed to be located beneath the pleura, with a sharp angle with the mediastinum, indicating that the lesion does not originate from the mediastinum. No enlarged lymph nodes are observed in the mediastinum or axilla, and there are no abnormal changes in the bones. Enhanced CT scans in arterial and venous phases show no enhancement changes in the lesions. The patient has no abdominal symptoms, and relevant examinations were not performed. However, during the chest CT examination (Fig. 5), multiple low-density masses are observed anterior to both kidneys in the retroperitoneum, with CT values similar to those of the orbital and chest lesions, and no enhancement changes are observed after contrast administration.

Due to the patient’s concurrent lung and abdominal lesions, after consultation with relevant departments to exclude surgical contraindications, we proceeded with the removal of the orbital masses. The patient underwent general anesthesia with endotracheal intubation, induced by intravenous propofol (2 mg/kg) and maintained with sevoflurane (1.5–2%) in oxygen-air mixture. Intraoperative monitoring included ECG, SpO₂, and blood pressure management. The surgery involved bilateral incisions below the eyebrow and conjunctival incision below the right eye. During the procedure, it was found that the mass above the bilateral orbits was adherent to the superior orbital nerve, while the lesion in the lower right orbit was adherent to the inferior orbital nerve. Multiple masses of various sizes were removed through the incision below the right eyebrow, ranging from approximately 4 cm × 2 cm to 1 cm × 0.6 cm, and a mass approximately 3 cm × 1.5 cm was removed through the conjunctival incision below the right eye. Additionally, a mass approximately 4 cm × 2.5 cm was removed through the incision below the left eyebrow. Each mass was enveloped in thin fascia, and after complete separation and removal, upon incision, a light yellow viscous fluid was observed flowing out from the masses.

**Figure 6.**
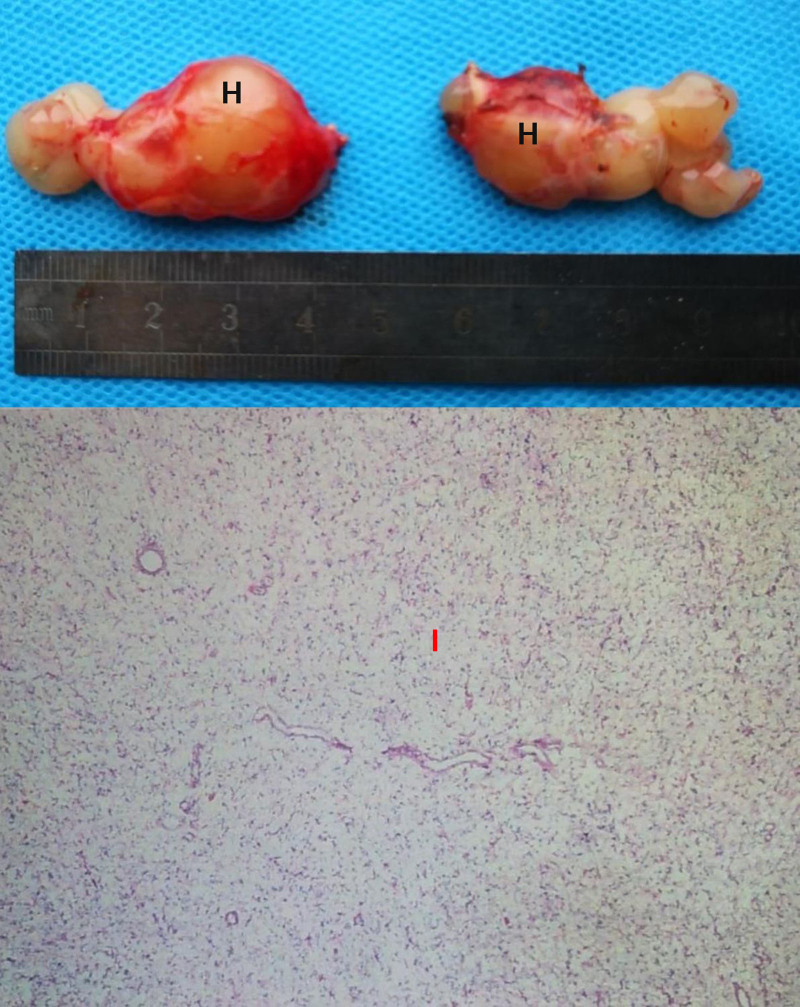
The picture shows the removal of the orbital tumor (H), and the pathological section is below (I). One week postoperatively, the patient’s limited eye movement has improved. Visual acuity is now 1.0 in the right eye and 0.8 in the left eye, representing an improvement compared to preoperative levels. Further treatment for the abdominal and pulmonary masses has not been initiated at this time.

Postoperative pathological examination (Fig. 6) indicates that the tissue morphology and immunohistochemistry of the orbital masses are consistent with mucinous tumors. Immunohistochemical staining of the tumor cells shows positive expression of Vimentin, weakly positive expression of S-100, positive expression of recombinant cluster of differentiation 34 (CD34), positive expression of GFAP, negative expression of CK, negative expression of actin (HHF35), and a Ki-67 proliferation index of 2%.

## 4. Outcomes

Pathological examination confirmed the diagnosis of mucinous tumors, with immunohistochemical profiling revealing a mesenchymal origin (Vimentin+, CD34+, GFAP+) and absence of epithelial differentiation (CK−, Actin−). The low Ki-67 index (2%) supported the benign nature of the lesions.

Clinically, the patient demonstrated rapid improvement post-surgery: Visual acuity improved from 0.5 to 0.8 in the left eye (right eye stable at 1.0). Ocular motility restored to full range without diplopia. Proptosis resolved completely, with no residual eyelid malposition or corneal exposure. Imaging follow-up at 3 and 6 months showed: No recurrence of orbital lesions. Stable pulmonary and retroperitoneal masses (unchanged in size/density), reinforcing the decision for surveillance.

Patient-reported outcomes included resolution of preoperative discomfort and restored quality of life. However, the patient remains under annual multidisciplinary review (ophthalmology, pulmonology, and radiology) to monitor potential late recurrence or progression of systemic lesions.

## 5. Discussion

This case of multifocal mucinous tumors involving the orbit, lungs, and retroperitoneum presents unique diagnostic and therapeutic challenges. The rarity of such widespread involvement raises important questions regarding disease pathogenesis, optimal management, and long-term outcomes. Below, we discuss the key findings, their clinical implications, and future directions for research and treatment.

### 5.1. Diagnostic considerations

The diagnosis of mucinous tumors relies on a combination of clinical, radiological, and histopathological findings. In our case, the bilateral orbital masses exhibited characteristic features on imaging, including homogeneous low-density lesions (14 HU on CT) and lack of contrast enhancement, consistent with prior reports.^[4]^ However, the presence of synchronous pulmonary and retroperitoneal lesions necessitated a broad differential diagnosis.

A critical step was excluding immunoglobulin G4 (IgG4)-related disease, which can mimic mucinous tumors radiologically. The absence of elevated serum IgG4 levels and typical histopathological findings (e.g., IgG4+ plasma cells) helped rule out this possibility. Additionally, cystic lymphangiomas – though sharing some imaging features – are typically seen in pediatric populations and exhibit even lower CT density values (<10 HU; Table 1). Neurofibromas, another consideration, usually demonstrate higher CT density (35–60 HU) and variable enhancement patterns. Our case underscores the importance of integrating clinical context with imaging and laboratory data to arrive at an accurate diagnosis^.[5]^

**Table 1:** Differential diagnosis of various types of orbital cysts.

Condition	Clinical presentation	Predominant site	Border	CT scan	CT enhancement	Ultrasound	MRI scan
Dermoid cyst	Painless proptosis	Superior to orbit	Clear	Irregular, negative	Ring-like	Uneven	T1: wall and T2: fluid, long T1, long T2 with fat present
Mucous cyst	Chronic sinusitis, headache, visual impairment	Upper nasal orbit	Clear	Uniform	Absent	Low	T2: Slightly long T1, long T2, or equal T1, long T2
Parasitic cyst (hydatid)	History of endemic area, consumption of undercooked meat	Within or adjacent to extraocular muscles	Clear	Uniform, may contain calcifications	Ring-like	None, may contain punctate echoes	Long T1, long T2 with low signal ring in the wall
Hematoma	Trauma, bleeding tendency, spontaneous, localized orbital vascular lesions	Subperiosteal or orbital connected to the orbital wall	Clear	Uniform, may have calcifications	Absent	Low	Acute: equal T1, equal T2; subacute: short T1, long T2; chronic: long T1, long T2
Cavernous hemangioma	Painless proptosis	Within extraocular muscles	Clear	Uniform	Progressive	Strong and evenly distributed	Equal T1, mixed T2
Cystic lymphangioma	Infants, proptosis, eyelid swelling, transparent conjunctival masses	External to extraocular muscles, eyelids	Unclear	Uniform	Absent	Uniform, cystic areas with low density	Equal T1, slightly long T2
Schwannoma	Progressive proptosis	Upper orbit	Clear	Uniform, cystic areas with low density	Clear	Low	Equal T1, mixed T2
Neurofibroma	Localized or widespread, eyelids, orbital soft tissues, brain, temporal region, skin, bones, viscera	Widespread	Clear (localized) or unclear (widespread)	Uniform or nonuniform	Mild	Low	Equal T1, slightly long T2
IgG4-related eye disease	Eyelid swelling without obvious cause, proptosis, periorbital tissue enlargement, dry eyes	Extraocular muscles, lacrimal glands, eyelids, orbital fat, superior and inferior orbital nerves, inferior orbital fissure and pterygopalatine fossa, extrinsic ocular glands such as parotid and submandibular glands	Unclear	Uniform (lymphoid proliferation)	Obvious	Low, uneven	Equal T1, slightly long T2

CT = computed tomography, IgG4 = immunoglobulin G4, MRI = magnetic resonance imaging.

### 5.2. Therapeutic approach and outcomes

The management of multifocal mucinous tumors remains controversial due to their rarity. For our patient, we adopted a staged approach: Surgical resection of the symptomatic orbital lesions to relieve mass effect and preserve vision. Active surveillance for the asymptomatic pulmonary and retroperitoneal lesions, given their indolent behavior and the risks of extensive surgery.

This strategy aligns with emerging evidence suggesting that asymptomatic extraorbital lesions may not require immediate intervention postoperatively, the patient showed significant improvement in visual acuity and ocular motility, supporting the role of surgery in symptomatic cases. However, the optimal management of unresectable or recurrent disease remains uncertain, highlighting the need for further research into systemic therapies.

### 5.3. Pathogenetic insights

The multifocal nature of this case raises intriguing questions about disease origin. Two hypotheses may explain the widespread involvement:

Embryonic remnant theory: The tumors may arise from dispersed remnants of embryonic mucoid tissue, as suggested by their distribution along developmental migration pathways.
Field defect hypothesis: A systemic abnormality in mesenchymal precursor cells could predispose to synchronous tumor development at multiple sites.

Immunohistochemical findings (CD34+/GFAP+) support a mesenchymal origin, but molecular studies are needed to explore potential genetic drivers.^[6]^

#### 5.3.1. Clinical significance and limitations

This case contributes to the literature in several ways: it challenges the traditional view of orbital mucinous tumors as strictly localized lesions. It provides imaging benchmarks (e.g., CT density < 20 HU) to aid future diagnoses. It validates a conservative approach for asymptomatic systemic involvement.

However, our study has limitations: The follow-up period (18 months) is insufficient to assess long-term recurrence risk. Molecular profiling was not performed, leaving open questions about genetic mechanisms. The single-case design limits generalizability.

### 5.4. Study limitations

While this case provides valuable insights, several limitations must be acknowledged. First, the single-case design restricts generalizability, and multicenter collaborations are needed to validate our observations. Second, the 18-month follow-up period is insufficient to assess long-term recurrence risks or the natural history of unresected systemic lesions. Third, molecular profiling (e.g., genetic mutations) was not performed, leaving potential pathogenic drivers unexplored. Lastly, the absence of standardized protocols for multifocal mucinous tumors necessitates cautious interpretation of our conservative approach to extraorbital lesions.

### 5.5. Future directions

To address these gaps, we recommend: establishing a multicenter registry to collate data on rare orbital tumors. Exploring targeted therapies, such as mammalian target of rapamycin inhibitors, for unresectable or recurrent disease.^[7]^ Investigating serum biomarkers (e.g., mucin-derived peptides) for noninvasive monitoring.

## 6. Conclusion

This case highlights the diagnostic and therapeutic complexities of multifocal mucinous tumors. Key takeaways include: multidisciplinary collaboration is essential for accurate diagnosis. Surgery remains first-line for symptomatic orbital lesions, while asymptomatic systemic disease may be managed conservatively. Long-term surveillance is crucial given the potential for recurrence. Future studies should focus on molecular characterization and the development of standardized treatment protocols for this rare condition.

## Author contributions

**Data curation:** Wen Gao.

**Investigation:** Wen Gao, Zhao Li.

**Resources:** Zhao Li.

**Validation:** Wen Gao.

**Writing – original draft:** Wen Gao.

**Writing – review & editing:** Zhao Li.
